# Seasonal Variation in Gut Microbiota of the Wild Daurian Ground Squirrel (*Spermophilus dauricus*): Metagenomic Insights into Seasonal Breeding

**DOI:** 10.3390/ani13132235

**Published:** 2023-07-07

**Authors:** Fengcheng Song, Shubao Ma, Yujiao Zhang, Xiaoying Yang, Haolin Zhang, Yingying Han, Yuning Liu, Fuli Gao, Zhengrong Yuan

**Affiliations:** College of Biological Sciences and Technology, Beijing Forestry University, Beijing 100083, China; nkjmkksfc218@bjfu.edu.cn (F.S.); msb2714442566@bjfu.edu.cn (S.M.); zyujiao@bjfu.edu.cn (Y.Z.); xiaoying_yang@bjfu.edu.cn (X.Y.); haolinzhang@bjfu.edu.cn (H.Z.); thinkinghyy@bjfu.edu.cn (Y.H.); yuningliu9@bjfu.edu.cn (Y.L.); fuligao@bjfu.edu.cn (F.G.)

**Keywords:** gut microbiota, wild Daurian ground squirrels, metagenome sequencing, seasonal breeding

## Abstract

**Simple Summary:**

Wild Daurian ground squirrels (*Spermophilus dauricus*) breed only a few months out of the year, a behavior known as seasonal breeding. Despite the gut microbiota being an essential “organ” of animals, little is understood about how they relate to seasonal breeding. In the present investigation, metagenomic sequencing techniques were employed to examine the diversity of gut microbiota in wild Daurian ground squirrels across different breeding seasons. The findings indicate notable variations in the gut microbiota’s structure and function among wild Daurian ground squirrels during different seasons. This study may provide an in-depth discussion of how seasonal reproduction affects gut microbes and aid in analyzing how changes in gut microbes act on the host. This study could provide new insights into the seasonal reproductive behavior of animals as well as a new theoretical basis for the study of gut microbiology.

**Abstract:**

The *Spermophilus dauricus,* the wild Daurian ground squirrel, is known to exhibit seasonal breeding behavior. Although the importance of gut microbiota in animal digestion, metabolism, and immunity is well-established, the correlation between gut microbiota and seasonal breeding in this species remains inadequately explored. In the present study, using metagenomic sequencing technology, the compositions and functions of the gut microbiota of wild Daurian ground squirrels in different breeding seasons were explored. The dominant gut microbial phyla were Firmicutes and Bacteroidetes. The Firmicutes were predominant in the breeding season, whereas Bacteroidetes were predominant in the non-breeding season. At the genus level, *Lactobacillus* accumulated during the breeding season, whereas *Odoribacter* and *Alistipes* increased during the non-breeding season. GO (Gene Ontology) and KEGG (Kyoto Encyclopedia of Genes and Genome) annotations indicated that genes in gut samples were highly associated with metabolic functions. The differential expression gene analysis showed that genes related to the phosphotransferase system, cysteine, and methionine metabolism were highly expressed during the breeding season, whereas the non-breeding season upregulated genes were enriched in starch and sucrose metabolism and bacterial chemotaxis pathways. In conclusion, this study could provide a reference for investigating gut microbiota in seasonal breeding animals and offer new insight into gut microbial function.

## 1. Introduction

The gut microbiota is defined as all microorganisms in the gastrointestinal tract of animals [1]. It comprises bacteria, viruses, fungi, and other microorganisms, with bacteria being the most abundant [2]. The gut microbiota is essential for human health, affecting many physiological functions, including metabolism [3,4] and immune system modulation [5]. One major function of the gut microbiota is to aid in the digestion of food. By converting indigestible carbohydrates and fiber into short-chain fatty acids (SCFAs), it provides the intestinal cells with a crucial source of energy [6]. The composition and abundance of gut microbiota are influenced by a combination of endogenous factors related to the host organism and exogenous environmental factors [7,8,9]. For example, gut microbiota showed significant differences between people who consumed more protein and those who consumed more carbohydrates [10]. In addition, the relationship between gut microbiota and reproduction is gaining attention. Gut microbiota can affect the reproductive capacity of animals by releasing hormones such as androgens [11,12,13] and estrogens [14,15]. A lack of normal gut microbiota leads to abnormal formation of the blood–testis barrier (BTB) in male mice [16]. It has also been suggested that polycystic ovary syndrome (PCOS) in women has a significant relationship with gut microbiota [17]. In a nutshell, the gut microbiota is vital for maintaining the health of the host and ensuring that the host can properly carry out its reproductive activities.

The wild Daurian ground squirrel (*Spermophilus dauricus*) has been extensively studied for their seasonal breeding behavior. During the breeding season (April to May), wild Daurian ground squirrels conduct breeding activities, whereas, during the non-breeding season (June to the following March), they do not [18]. Seasonal breeding is mainly influenced by photoperiod [19,20]. The mammalian pineal gland senses photoperiodic changes and translates such changes into chemical signals in the form of secreted melatonin [21]. Mammals use changes in the timing of melatonin secretion at night as a signal to regulate reproductive cycle changes via the hypothalamic–pituitary–gonadal (HPG) axis [22]. The gonadotropin-releasing hormone (GnRH) neurons in the hypothalamus secrete GnRH, which in turn acts on the pituitary gland through the pituitary portal system, and the pituitary gland responds to GnRH stimulation by secreting follicle-stimulating hormone (FSH) and luteinizing hormone (LH) [23,24]. In addition, kisspeptin [25], thyroid hormone [26], and gonadotropin inhibitory hormone (GnIH) [27] can also be involved in regulating the seasonal reproduction of animals.

There are some studies on the seasonal breeding behavior of wild Daurian ground squirrels. Our previous studies showed that the expression levels of sex hormone receptors and other hormones were seasonally different in the hypothalamus, testes, ovaries, and other HPG axis-related organs of wild Daurian ground squirrels [28,29,30,31,32,33,34]. Wild Daurian ground squirrels also show some seasonal differences in their gut [35]. Previously, we found that the important SCFA receptors G-protein-coupled receptor 41 (GPR41) and G-protein-coupled receptor 43 (GPR43) in the wild Daurian ground squirrels were seasonally expressed in the colon [36]. In prior research, we used 16s rRNA sequencing to initially analyze the gut microbiota composition of wild Daurian ground squirrels [37], however, we did not use metagenomic sequencing to examine this issue. Comparing with the 16S rRNA sequencing method, metagenomic sequencing has higher resolution, can be identified at the species level, and has the advantage of obtaining more genetic information through gene prediction [38]. Therefore, metagenomic analysis of their gut microbiota is necessary for providing more evidence that can explain the complex interaction between seasonal breeding and gut microbiota.

Using metagenomic technology, this study investigated the variation between breeding seasons in α-diversity, β-diversity, species composition, and functional gene expression in the cecum microbiota of male wild Daurian ground squirrels. The primary objectives of this research were to deepen our understanding of the changes occurring within the gut microbiota of wild Daurian ground squirrels across different breeding seasons, to elucidate the response mechanisms of the gut microbiota to seasonal change, and to identify potential pathways by which the gut microbiota modulate seasonal breeding. By employing rigorous analytical techniques and statistical methods, the present study provides valuable insights into the intricate interplay between gut microbiota and seasonal breeding in wild Daurian ground squirrels.

## 2. Materials and Methods

### 2.1. Experimental Animal Acquisition and Sample Collection

We obtained adult male wild Daurian ground squirrels in April (breeding season, B, *n* = 5) and June (non-breeding season, NB, *n* = 5). All experimental animals were captured in the wild at Bashang Grassland, Guyuan County, Hebei Province, China (40.83° N 114.88° E). When the animals were brought to the laboratory, they were directly anesthetized via inhalation of CO_2_ and executed, and they were then immediately dissected using sterile laboratory instruments to collect the cecum contents. Samples were collected in sterilized 5 mL centrifuge tubes and stored at −80 °C to carry out subsequent experimental investigations. All animal experiments were approved by the Institutional Review Board (or Ethics Committee) of Beijing Forestry University and the Department of Agriculture of Hebei Province, China (JNZF11/2007).

### 2.2. DNA Extraction and Metagenomic Sequencing

DNA was extracted according to the instructions of the TIANamp Stool DNA Kit (TIANGEN Biotech Co., Ltd., Beijing, China). Samples were extracted and analyzed individually (with samples of 100 ng used). DNA was quantified for purity using a NanoDrop 8000 UV-Vis spectrophotometer (Thermo Scientific, Wilmington, DE, USA), and DNA integrity was examined using 1.0% agarose gel electrophoresis. The DNA libraries were constructed by sequentially fragmenting the DNA samples, PCR amplification, and library fragment size screening according to the instructions of the Enzymic Universal DNAseq Library Prep Kit (Kaitai Bio-Technology Co., Ltd., Hangzhou, China, more detailed information on this kit is shown in the Supplementary Methods). Volumes of 5 μL of the PCR products were taken for 1% agarose gel electrophoresis to determine whether the PCR samples were qualified. Then, the PCR products were purified using the AMPure XP system (Agencourt Bioscience Corporation, Beverly, MA, USA). The average library length was evaluated using the Agilent 2100 Bioanalyzer system (Agilent, Santa Clara, CA, USA), whereas the quality levels of the libraries were assessed using a Qubit fluorometer (Thermo Fisher Scientific, Waltham, MA, USA). Finally, the qualified libraries were sequenced on the Illumina Novaseq 6000 platform (Illumina, San Diego, CA, USA), and 150 bp paired-end reads were generated for metagenomic sequencing.

### 2.3. Data Processing, Species Composition Analysis, and Database Annotation

Using raw sequencing data, low-quality regions were removed from the subsequent analysis to ensure clean data. The specific data quality control steps are shown in the Supplementary Methods. The species classification of the sequenced reads was performed using MetaPhlAn V2.0 [39]. Based on high-quality sequences, the metagenomic sequences were assembled using SOAPdenovo V2.04 [40], and then the shorter (<200 bp) sequences were filtered out to obtain scaffold sequences. Gene prediction was performed using MetaGeneMark V3.25 [41], and results with coding frames smaller than 100 bp were filtered out. The sequences were then clustered using CD-HIT V4.8.1 [42], and if the similarity between two sequences was greater than 95% and covered 90% of the region of the shorter one [43], the sequences were clustered as one sequence. The clustering result is the non-redundant (NR) gene set. Blastp V2.12.0 [44] was used for comparison with public databases for the annotation of non-redundant genes (identity ≥ 30%, e-value ≤ 1 × 10^−5^). Databases used include the Gene Ontology (GO) database [45] and the Kyoto Encyclopedia of Genes and Genomes (KEGG) database [46].

### 2.4. Differentially Expressed Gene Analysis

Based on the counted numbers of genes, the differentially expressed genes (DEGs) of the breeding and non-breeding seasons were analyzed using DESeq2 [47]. Genes satisfying |log2(Fold change)| > 1 and Q value ≤ 0.05 were assigned as DEGs. To obtain the KEGG terms significantly enriched in the differential genes, we used a hypergeometric test. The pathways with *p* < 0.05 for the test were defined as the KEGG terms significantly enriched in differential genes. This process was implemented with the R package clusterProfiler V3.12 [48].

### 2.5. Statistical Analysis

To examine whether there were seasonal differences in the diversity of gut microbial communities, we analyzed their α-diversity and β-diversity. α-diversity was used to illustrate the average species diversity of a sample, including the richness and evenness of the species. β-diversity, on the other hand, was dedicated to the comparison between different samples, and it used the change in abundance between different samples to calculate the inter-sample distance, thereby reflecting whether there are significant microbial community differences between samples. In this study, the Shannon index and Simpson index were used to describing α-diversity, and the Bray–Curtis distance was used to illustrate β-diversity. Mothur V1.41 [49] was used to calculate the Shannon index, Simpson index, and Bray–Curtis distance. To test whether α-diversity was significant between the two groups, we performed a *t*-test (*p* < 0.05). In addition, non-parametric analysis of similarity (ANOSIM) was used to test whether there were statistical differences in β-diversity between groups, and Principal Coordinate Analysis (PCoA) was used to present the differences between groups based on the Bray–Curtis distance matrix. The *t*-test was used to obtain gates and genera that differed significantly between groups, and the results were visualized using an extended error bar plot. This process was performed using STAMP V2.1.3 [50]. Linear Discriminant Analysis Effect Size (LEfSe) [51] was utilized to find more significantly different biomarkers (LDAScore > 2, *p* < 0.05). This work was done on the website (https://huttenhower.sph.harvard.edu/galaxy/, accessed on 19 July 2022.). The statistics of KEGG and GO annotation results were achieved by relying on base R V4.1.1. Species composition histograms, functional statistics plots, and bubble plots for differential enrichment analysis were created using the R package ggplot2 V3.1.0 [52], and species diversity analysis plots were created using the online website ImageGP [53]. This section is written with reference to an article on writing methods [54].

## 3. Results

A total of 847.686 million raw reads were obtained from wild Daurian ground squirrel cecum content samples, and 827.756 million clean reads were obtained after quality control, with an average length of 149.31 bp (Table S1). The metagenomic sequences were assembled based on high-quality sequences. The assembled genome size was 342,274,624 bp with 136,999 scaffold sequences (Table S2). Gene prediction obtained 549, 161 NR genes with an average length of 385.9 bp. The species accumulation curve was generated based on the identified species (Figure S1). Towards the end of the curve, a plateauing trend was observed, indicating that the sample size was sufficient.

### 3.1. Species Composition and Variation of the Gut Microbiota

**Figure 1:**
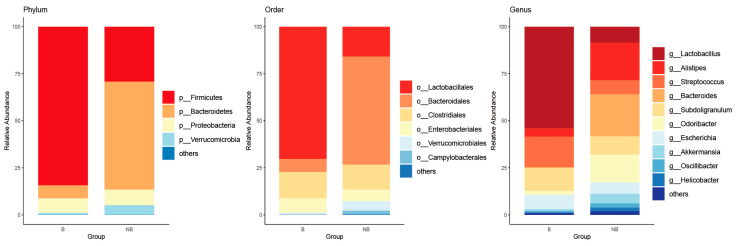
Composition of gut microbial species in wild Daurian ground squirrels. The bar stacks were plotted using the mean relative abundance at each taxonomic level (from left to right: phylum, order, and genus levels); the others are the set of all species with relative abundance <1%. B, breeding season; NB, non-breeding season.

Species composition analysis was performed based on the species taxonomic information obtained using MetaPhlAn2 (Table S3). The main species (relative abundance >1%) of the phylum, order, and genus were presented (Figure 1). The phylum Firmicutes and the phylum Bacteroidetes showed great dominance, with the sum of their relative abundances accounting for more than 80% of the gut microbiota, in both B and NB squirrels. Other dominant phyla were Proteobacteria and Verrucomicrobia. The main genera are *Lactobacillus, Alistipes*, *Streptococcus*, *Bacteroides*, *Subdoligranulum*, *Odoribacter*, *Escherichia*, *Akkermansia*, *Oscillibacter*, and *Helicobacter*, which accounted for more than 98% of the relative abundance of all genera.

**Figure 2:**
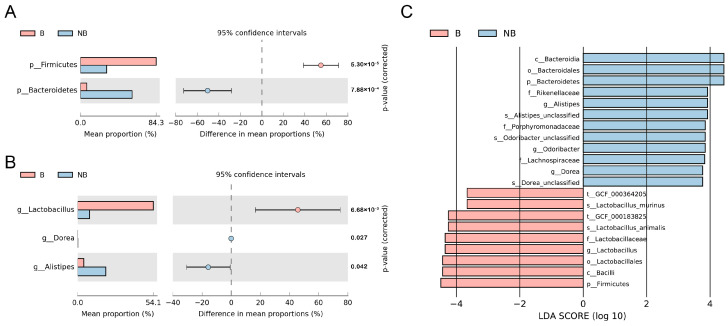
Variation in species composition of gut microbiota during different seasons. The extended error bar plot for significant variation in gut microbiota at the (**A**) phylum level and (**B**) genus level. (**C**) The LEfSe histogram displays the distribution of LDA values for biomarkers with significant differences (LDAScore > 2, *p* < 0.05). B, breeding season; NB, non-breeding season; LEfSe, Linear Discriminant Analysis Effect Size; LDA, Linear Discriminant Analysis.

Meanwhile, the species information was used to explore the differences in the gut microbial species composition of cecum samples from animals in different breeding seasons. The variation analysis was performed for different taxonomic levels (Figure 2A,B). At the phylum level, the two most represented phyla likewise showed significant seasonal differences (*p* < 0.01). The phylum Firmicutes was dominant in the cecum samples collected from breeding-season males, whereas the phylum Bacteroidetes was dominant in the non-breeding male cecum samples. In the case of differential genera (*p* < 0.05), *Lactobacillus* was richer in samples from breeding season males, whereas *Alistipes* and *Dorea* showed higher abundance in the non-breeding season samples.

LEfSe analysis provided further information on differential species composition (Figure 2C). The relative abundances of family Rikenellaceae, family Porphyromonadaceae, and genus *Odoribacter* were high during the non-breeding season, the relative abundances of class Bacilli and two species, *Lactobacillus animalis,* and *Lactobacillus murinus*, were high during the non-breeding season.

### 3.2. Diversity of the Gut Microbiota

**Figure 3:**
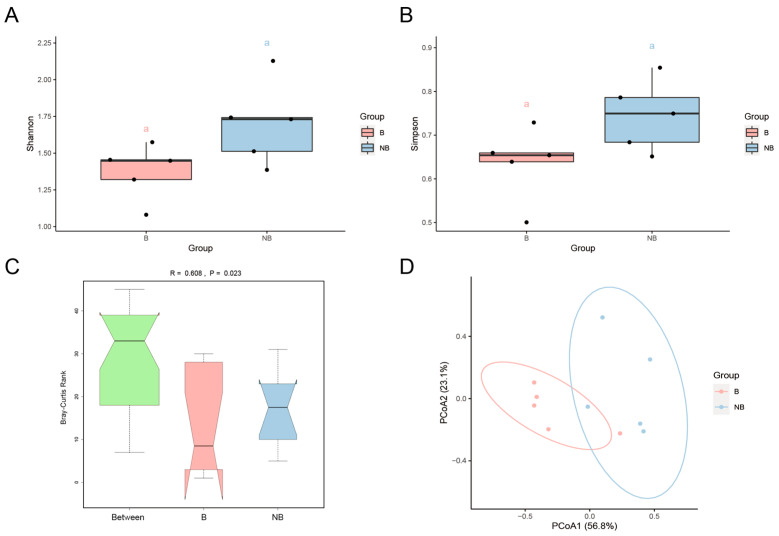
Analysis of gut microbial diversity during the breeding season (B) and the non-breeding season (NB). (**A**) The box plot of the Shannon index. (**B**) The box plot of the Simpson index. (Lowercase letters in the plots indicate the significant differences). (**C**) ANOSIM based on Bray–Curtis distance. (**D**) In the PCoA plots using Bray–Curtis distances, PCoA1 explained 56.8% and PCoA2 explained 23.1% of the total variation of the samples. B, breeding season; NB, non-breeding season; ANOSIM, Analysis of Similarity; PCoA, Principal Coordinate Analysis.

The results of alpha diversity analysis revealed no significant differences in the microbial communities of cecum samples collected during different breeding seasons, as determined by using both the Shannon index and the Simpson index (Figure 3A,B). These findings suggest that there is no remarkable variation in the abundance and distribution of gut microbiota between these two seasons. As for β-diversity, the results of ANOSIM showed an R-value greater than 0 (*p* < 0.05), indicating that the differences between the different groupings were significant and that the groupings were meaningful (Figure 3C). In addition, the confidence ellipses of the breeding season and non-breeding season samples were well-separated in the PCoA plot, demonstrating that the gut microbiota of these two groups differed significantly in terms of composition and abundance (Figure 3D).

### 3.3. Functional Annotation of the Gut Microbiota

**Figure 4:**
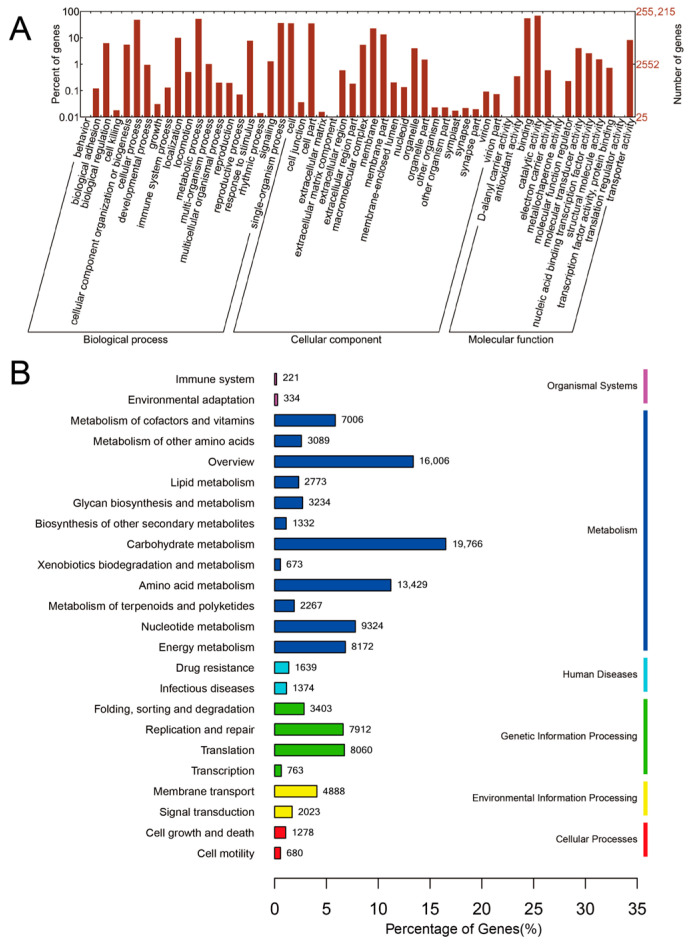
Functional statistical analysis of the gut microbiota of wild Daurian ground squirrels. (**A**) The bar plot of GO database annotations. (**B**) The annotated statistical plot of gut microbiota in KEGG. B, breeding season; NB, non-breeding season; GO, Gene Ontology; KEGG, Kyoto Encyclopedia of Genes and Genome.

The NR sequences obtained from gene prediction were annotated based on the GO and KEGG databases. A total of 255,215 genes were annotated based on the GO database, and 119,646 genes were annotated based on the KEGG pathway database. The annotation results were statistically analyzed to obtain functional enrichment in both databases (Figure 4). 

The statistical plot of the GO database is shown in Figure 4A. In terms of biological processes, the gut microbiota sequence of wild Daurian ground squirrels was annotated to the main functions of cellular processes, metabolic processes, biological regulation, rhythmic processes, responses to stimulus, reproductive processes, and single-organism processes. In terms of cellular components, the genes of the intestinal microbiota were mainly enriched in cells, membranes, and organelles. In molecular function, the genes of the gut microbiota were enriched in binding, catalytic activity, transcription factor activity protein binding, transporter activity, molecule transducer activity, and transporter activity functions. 

Regarding the KEGG database, the pathway categories of genes of the wild ground squirrel gut microbiota include metabolism, organismal systems, genetic information processing, human diseases, cellular processes, and environmental information processing, the most important of which is metabolism, including carbohydrate metabolism, metabolism of cofactors and vitamins, amino acid metabolism, energy metabolism, and nucleotide metabolism.

### 3.4. Analysis and Enrichment of Differentially Expressed Genes

**Figure 5:**
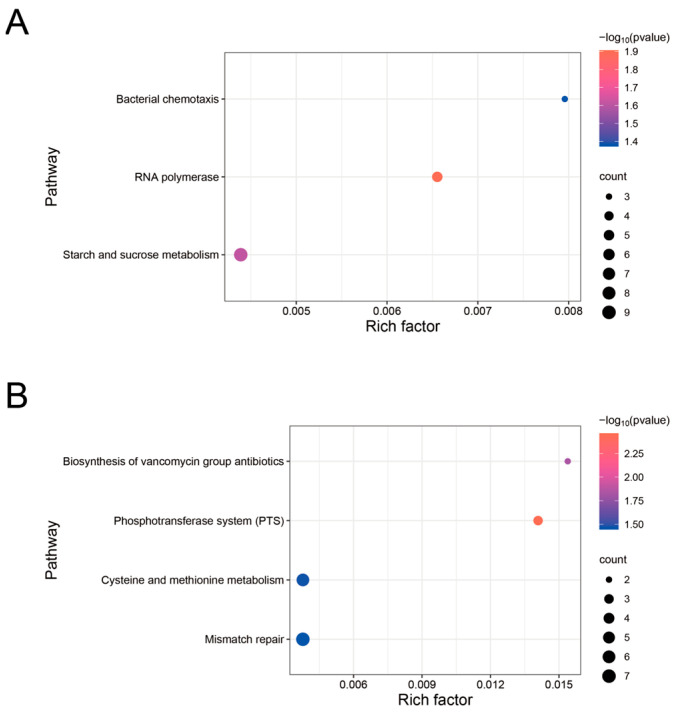
The bubble plot of KEGG differential enrichment (“Rich factor” = the number of differential genes enriched to the pathway/the number of background genes in the pathway). (**A**) Pathways that were highly expressed in the non-breeding season. (**B**) Pathways that were highly expressed in the breeding season.

A total of 1574 differentially expressed genes satisfying |log2(fold change)| > 1 and Q value ≤ 0.05 were found. Among them, 656 genes were upregulated and 918 genes were downregulated in the non-breeding season in comparison to the breeding season (Figure S2). Differentially expressed gene enrichment analysis was performed by applying phyper to identify KEGG pathways that were significantly enriched in differential genes compared to all gene backgrounds (*p* < 0.05). The results showed that there were three pathways with increased expression during the non-breeding season, namely RNA polymerase, starch and sucrose metabolism, and bacterial chemotaxis (Figure 5A), whereas four pathways were upregulated during the breeding season, namely. phosphotransferase system (PTS), biosynthesis of vancomycin group antibiotics, cysteine and methionine metabolism, and mismatch repair (Figure 5B).

## 4. Discussion

Gut microbiota is important for animals to maintain normal life activities. At the phylum level, Firmicutes and Bacteroidetes dominated the gut content samples of breeding and non-breeding wild Daurian ground squirrels, which is similar to other rodents such as arctic ground squirrels (*Urocitellus parryii*) [55], wild wood mice (*Apodemus sylvaticus*) [56], and North American red squirrels (*Tamiasciurus hudsonicus*) [57]. In fact, in many other wild animals, such as the Indo-Pacific humpback dolphin (*Sousa chinensis*) [58], snub-nosed monkey (*Rhinopithecus* spp.) [59], and other mammals [60], the dominant phyla in the gut microbiota are also Firmicutes and Bacteroidetes. α-diversity and β-diversity are both important metrics for describing community diversity. α-diversity is mainly used to measure the richness and the evenness of species within a community, whereas β-diversity measures the differences in the number and distribution of each species compared among communities [61]. One study found that the α-diversity of gut microbiota in Siberian chipmunks (*Tamias sibiricus*) increased significantly during hibernation [62]. However, the α-diversity of wild Daurian ground squirrels’ gut microbiota did not show remarkable changes associated with seasonal breeding, suggesting that the change in breeding status did not affect the overall richness of their gut microbiota. In terms of β-diversity, there were remarkable differences in microbial communities between the samples collected from males in breeding and non-breeding seasons, indicating significant seasonal variation in the gut microbial composition of wild Daurian ground squirrels. It has been shown that the pineal gland of Siberian hamsters (*Phodopus sungorus*) regulates the composition of gut microbiota [63] and that seasonal breeding behavior is similarly regulated by this organ. Therefore, it is possible that changes observed in the gut microbial β-diversity in wild Daurian ground squirrel samples collected from breeding and non-breeding individuals were a response to reproductive activity.

The diet structure of wild Daurian ground squirrels changes from the breeding season to the non-breeding season. Wild Daurian ground squirrels consume a greater proportion of protein and fat during the breeding season, yet wild Daurian ground squirrels in the non-breeding season consume a large total amount of food and weigh more to store fat and survive hibernation [64]. Analysis of the differences in gut microbiota showed that at the phylum level, Firmicutes dominated during the breeding season, whereas Bacteroidetes dominated during the non-breeding season. In a study of another animal with seasonal breeding behavior, the plateau pika (*Ochotona curzoniae*), it was similarly shown that the relative abundance of Bacteroidetes in the gut microbiota was significantly higher during the non-breeding season than during the breeding season [65]. The Firmicutes/Bacteroidetes (F/B) ratio was significantly higher during the breeding season, which may indicate that the gut microbiota of these two phyla is closely associated with the seasonal reproduction of rodents. In previous studies on human gut microbiota, an elevated F/B ratio was often highly correlated with the development of obesity [66,67]. The Firmicutes in the gut have better efficiency in the breakdown of lipids as well as carbohydrates, which is more conducive to the absorption of nutrients from the food by the host [68]. Similarly, it has been shown that excessive energy intake leads to the proliferation of Firmicutes in the guts of rodents [69], thereby demonstrating that an increase in the number of Firmicutes in the gut facilitates energy uptake. In contrast, Bacteroidetes is widely considered to play an important role in the digestion of dietary fiber due to the ability of Bacteroidetes to break down a variety of phytoglycans, including cellulose, hemicellulose pectin, and other major components of plant cell walls [70]. Hence, it is speculated that the elevated F/B ratio observed during the breeding season may be to allow the host to better obtain nutrients from the gut for the high energy consumption required for reproduction. The decrease in the F/B ratio during the non-breeding season, on the other hand, maybe to better adapt to the high-fiber diet structure of the non-breeding season, quickly acquire and store energy, and prepare for hibernation.

At the genus level, the genus *Lactobacillus* was significantly elevated during the breeding season, whereas the non-breeding season was enriched with *Alistipes*, *Odoribacter,* and *Dorea*. *Lactobacillus* is an important member of the gut microbiota, which can ferment carbohydrates to produce lactic acid, and it is a widely recognized probiotic [71]. LEfSe analysis showed that two species, *Lactobacillus murinus,* and *Lactobacillus animalis*, were more abundant in the breeding season. Many species in *Lactobacillus* can help their hosts digest unmetabolizable nutrients by regulating the synthesis of B vitamins (B2, B9, and B12) [72]. The dietary composition of breeding wild Daurian ground squirrels is more complex than that of non-breeding squirrels, and reproductive activity requires an efficient energy supply. The elevated level of *Lactobacillus* helps to increase the efficiency of energy metabolism for the host. In addition, bacteria from *Lactobacillus* can attenuate testicular dysfunction in male mice by affecting the secretion of hormones (e.g., GnRH) associated with the HPG axis [73], which is critical for regulating seasonal breeding behavior. Several studies have also shown that *Lactobacillus* can improve spermatogenesis and enhance sperm quality in animals [74,75,76]. This is consistent with our previous findings that wild Daurian ground squirrels produce large and active sperm during the breeding season and no sperm during the non-breeding season [77,78]. In summary, it was hypothesized that *Lactobacillus* may be implicated in the regulation of seasonal breeding of wild Daurian ground squirrels by influencing their metabolism, HPG axis, and reproductive physiology. Among the genera with high abundance during the non-breeding season, *Alistipes* and *Odoribacter* were reported to be closely associated with the production of acetate and propionate [79]. One study claimed that the acetic acid produced by gut microbiota increased brain stimulation of the vagus nerve, promoting elevated appetite as well as fat gain [80]. It is possible that the *Alistipes* and *Odoribacter* enriched in the non-breeding season could help wild Daurian ground squirrels increase their appetite and store fat through this pathway.

The functional analysis involved showed that the gut microbiota of wild Daurian ground squirrels was mainly enriched in metabolism-related pathways. The most important function of gut microbiota is their metabolic function [81], which is well-verified by the functional enrichment results. The differentially expressed genes KEGG enrichment results revealed several pathways that were significantly altered during the breeding versus the non-breeding seasons. High expression in the pathways of PTS, biosynthesis of vancomycin group antibiotics, cysteine, and methionine metabolism was found during the breeding season. The PTS is a complex device present in bacteria that is responsible for the uptake and phosphorylation of large amounts of carbohydrates during bacterial energy transport [82]. Bacteria of *Lactobacillus* can ferment carbohydrates into lactic acid via the PTS [83] and provide sufficient energy for the breeding activities of wild Daurian ground squirrels. Breeding wild Daurian ground squirrels consume more insects and have increased protein intake, which may be associated with high expression of cysteine and methionine metabolism. A study has shown that vancomycin treatment leads to an increase in *L. murinus* in the intestine of mice [84]. The breeding season gut microbiota in this study was exactly enriched with the enrichment of biosynthesis of the vancomycin group antibiotics pathway and had a large amount of *L. murinus*, verifying this point. The pathways enriched by non-reproductive upregulated genes are starch and sucrose metabolism and bacterial chemotaxis. The enrichment of the starch and sucrose metabolism pathway implies that gut microbiota may help non-breeding wild Daurian ground squirrels metabolize large amounts of carbohydrates.

Before this study, we also performed a metagenomic analysis of the gut microbiota of another seasonal breeding animal, the muskrat (*Ondatra zibethicus*) [85]. Comparing these two results, we found that their gut microbiota had the opposite ratios of Firmicutes to Bacteroidetes concerning the season. This result was mainly due to the difference in their feeding habits and breeding season. However, we found that they showed similarity in terms of KEGG pathway enrichment. All of them showed a high correlation with starch and sucrose metabolism and bacterial chemotaxis during the non-breeding season, whereas during the breeding season, they were both enriched for pathways associated with amino acid metabolism. These pathways may have an important connection with seasonal breeding behavior.

In a nutshell, in the present study, a metagenomic approach was used to explore seasonal variation in gut microbiota in wild Daurian ground squirrels. This study explored in greater depth how gut microbiota respond to changes in the reproductive state of animals and how they in turn regulate seasonal breeding behavior. This study may provide new references for researchers in related fields when studying the reproductive behavior of wild Daurian ground squirrels or other seasonally breeding animals. Combined with previous studies, it could provide a new reference and theoretical basis for researchers in related fields when studying the reproductive behavior of wild Daurian ground squirrels or other seasonal breeding animals, and gut microbiology could be considered as a new starting point for explaining the mechanisms of seasonal breeding.

## 5. Conclusions

The present study provides a new metagenomic insight into the relationship between gut microbiota and seasonal breeding. Significant seasonal variation was found in the species composition and function of gut microbiota in wild Daurian ground squirrels. The abundance of Firmicutes increased during the breeding season, whereas Bacteroidetes was enriched during the non-breeding season. Genus *Lactobacillus* was enriched during the breeding season, whereas the genus *Odoribacter* and genus *Alistipes* were enriched during the non-breeding season. Wild Daurian ground squirrels’ gut microbiota were highly enriched in metabolism-related pathways. Genes associated with the PTS, biosynthesis of vancomycin group antibiotics, and cysteine and methionine metabolism pathways were increased during the breeding season, whereas genes upregulated during the non-breeding season were enriched in starch and sucrose metabolism and bacterial chemotaxis pathways. Gut microbiota may modulate seasonal breeding in wild Daurian squirrels by affecting their metabolism, HPG axis, and reproductive system functions.

## Data Availability

Not applicable.

## Supplementary Materials

The following supporting information can be downloaded at: https://www.mdpi.com/article/10.3390/ani13132235/s1, Figure S1: The species accumulation curves. Figure S2: The volcano map of gene expression between groups. Table S1: Raw data information from sequencing and clean data information after quality control. Table S2: Statistical results of genome assembly. Table S3: Abundance statistics for all taxonomic levels (phylum, class, order, family, genus, and species). Supplementary Methods: Detailed information on the Library Prep kit and raw data quality control procedure. References [86,87] are cited in the supplementary materials.
