# Supplementary material for: Seasonal Variation in Gut Microbiota of the Wild Daurian Ground Squirrel (Spermophilus dauricus): Metagenomic Insights into Seasonal Breeding
Source: Animals (Basel). 2023 Jul 7;13(13):2235. doi: 10.3390/ani13132235 (PMC10340016; doi:10.3390/ani13132235)
Supplement: Supplementary file 1 [file animals-13-02235-s001.zip › Supplementary Materials/Figure S1.pdf]

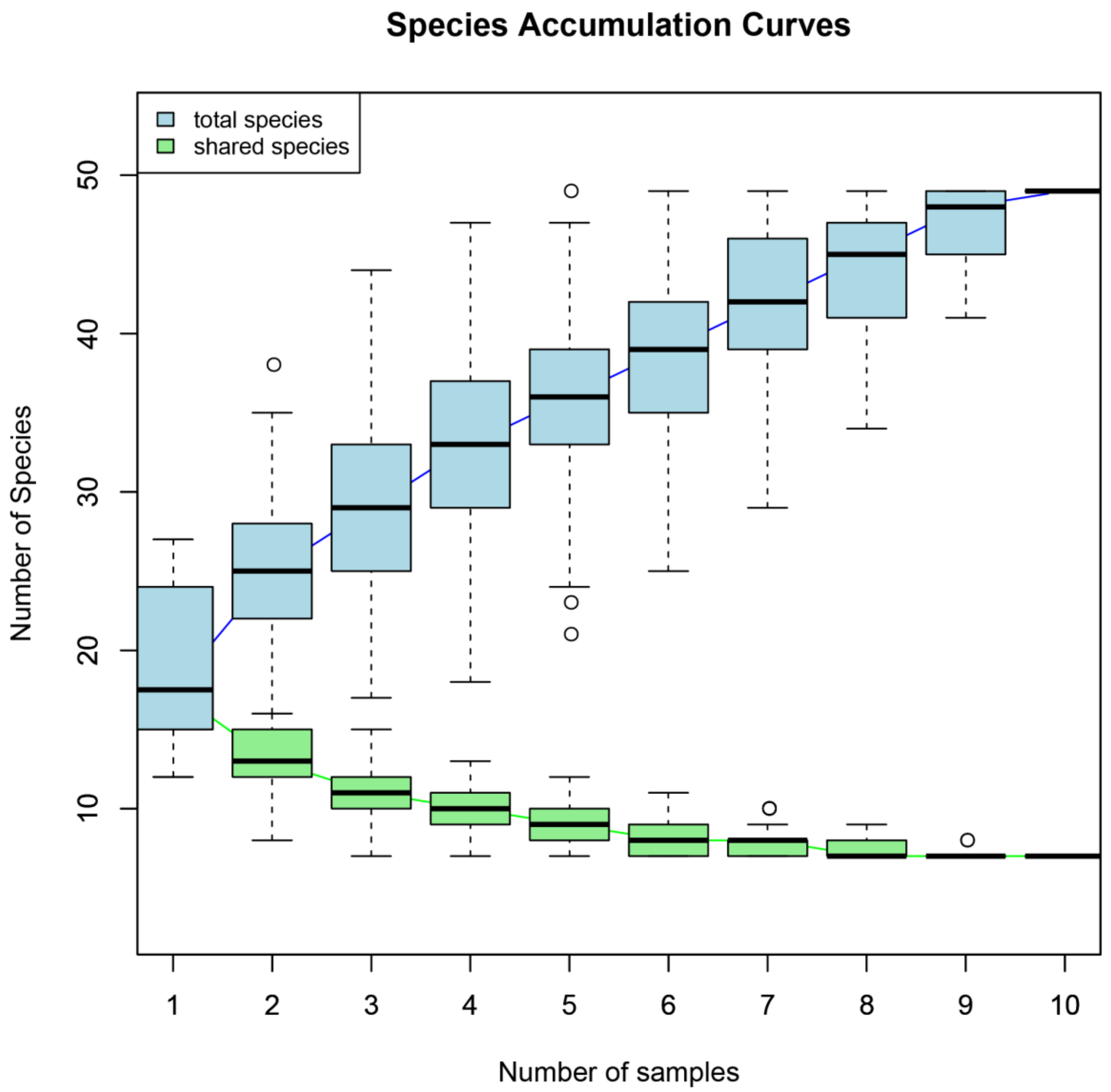

Figure S1 The species accumulation curves. The horizontal axis is the number of samples and the vertical axis is the number of total and shared species at different sample sizes (“○” represent outliers).
