# Supplementary material for: Seasonal Variation in Gut Microbiota of the Wild Daurian Ground Squirrel (Spermophilus dauricus): Metagenomic Insights into Seasonal Breeding
Source: Animals (Basel). 2023 Jul 7;13(13):2235. doi: 10.3390/ani13132235 (PMC10340016; doi:10.3390/ani13132235)
Supplement: Supplementary file 1 [file animals-13-02235-s001.zip › Supplementary Materials/Figure S2.pdf]

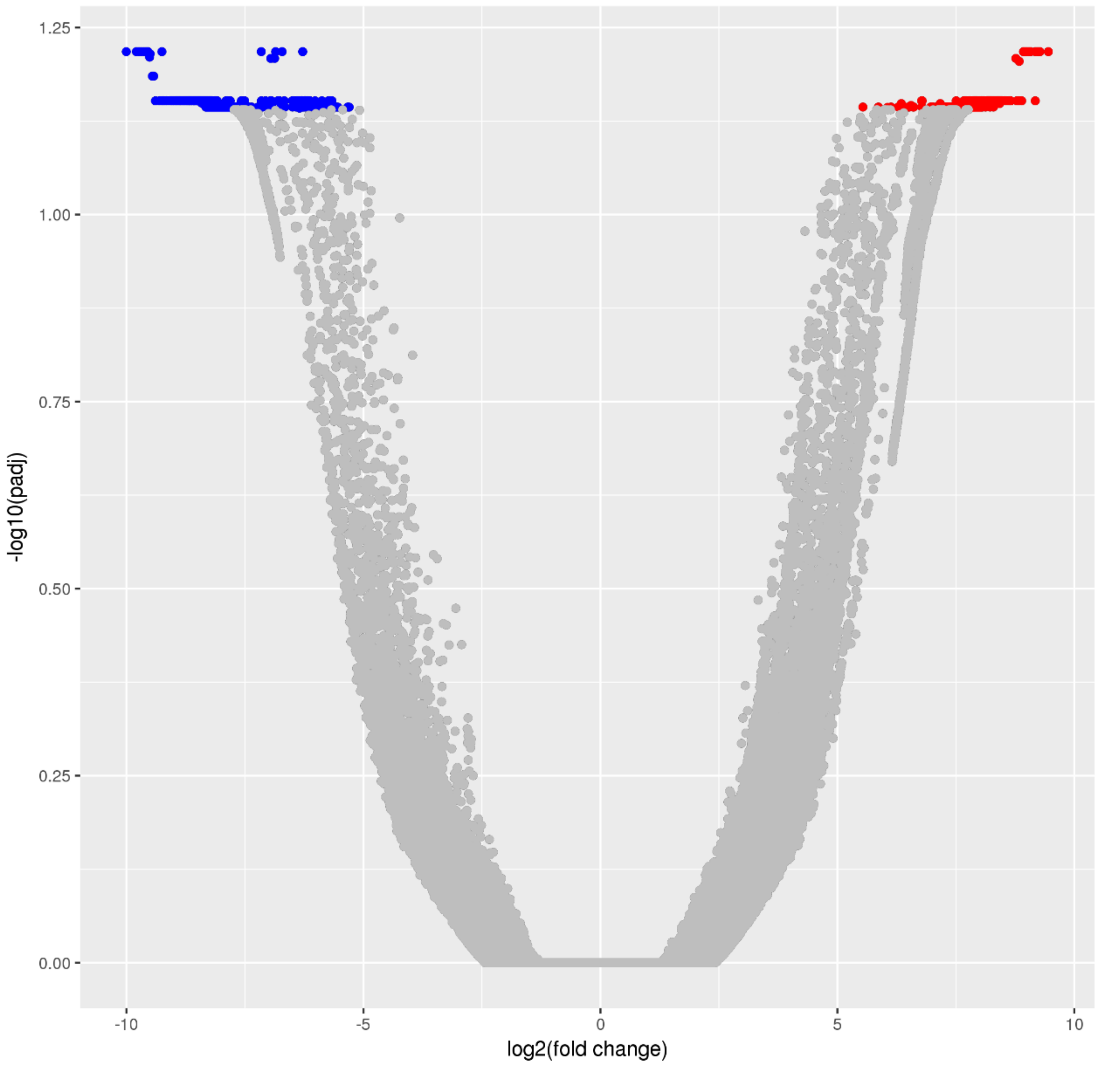

Figure S2 The volcano map of gene expression between groups. Horizontal coordinates indicate log2(fold change) of genes in the breeding and non-breeding seasons; vertical coordinates indicate negative log values of P-adjust values for gene expression changes. Red points represent genes that are down-regulated during the breeding season, and blue points represent genes that are up-regulated during the breeding season.
