# Supplementary material for: Seasonal Variation in Gut Microbiota of the Wild Daurian Ground Squirrel (Spermophilus dauricus): Metagenomic Insights into Seasonal Breeding
Source: Animals (Basel). 2023 Jul 7;13(13):2235. doi: 10.3390/ani13132235 (PMC10340016; doi:10.3390/ani13132235)
Supplement: Supplementary file 1 [file animals-13-02235-s001.zip › Supplementary Materials/Supplementary Methods.pdf]

Method 1. Content for Enzymic Universal DNAseq Library Prep Kit (Kaitai Bio-Technology Co., Ltd., Hangzhou, China)

Content	AT4107-01 (6 preps)
DNA frag Buffer	15 uL
DNA frag Enzyme	30 uL
DNA Ligation Buffer	24 uL
DNA Ligation Enzyme	6 uL
DNA Universal ADT	6 uL
2 x PCR Mix	150 uL
DNA-Universal Primer	12 uL

Method 2. Raw data obtained by sequencing using Illumina sequencing platform has a certain proportion of low-quality data. In order to ensure the accuracy and reliability of the results of subsequent analysis, the raw sequencing data need to be pre-processed by fastp[1], the specific processing steps are as follows:

(1) Remove the adapter sequence from sequencing reads;

(2) Scanning of sequencing reads by sliding window method, with a default scan window of 6 bp, and cutting off the portion of the read from the beginning of the window to the 3' termination when the average quality value in the window is below 20;

(3) Remove the reads containing more than 40% of unqualified bases (mass <= 15);

(4) Remove sequences less than 100bp in length;

(5) Remove sequences with more than 5% N content;

(6) Use Bowtie 2[2] to compare with host sequences and filter out reads that may be from the host.

References

1. Chen, S.; Zhou, Y.; Chen, Y.; Gu, J. fastp: an ultra-fast all-in-one FASTQ preprocessor. *Bioinformatics* **2018**, *34*, i884-i890, doi:10.1093/bioinformatics/bty560.
2. Langmead, B.; Salzberg, S.L. Fast gapped-read alignment with Bowtie 2. *Nature Methods* **2012**, *9*, 357-359, doi:10.1038/nmeth.1923.
